# 523. A Rapid, Objective Measure of the Host Response May Help Guide Efficient and Judicious ED Care for Sepsis

**DOI:** 10.1093/ofid/ofac492.578

**Published:** 2022-12-15

**Authors:** Hollis R O’Neal, Roya Sheybani, Henry T Tse, Ajay M Shah, Christopher B Thomas

**Affiliations:** Louisiana State University Health Sciences Center, Baton Rouge, Louisiana; CytoVale, Inc., San Francisco, California; CytoVale, Inc., San Francisco, California; CytoVale, Inc., San Francisco, California; Louisiana State University Health Sciences Center, Baton Rouge, Louisiana

## Abstract

**Background:**

Most cases of sepsis present to Emergency Departments (ED’s). Current guidelines stress ED clinicians to intervene before adequate, objective diagnostic and prognostic data are available, resulting in a ‘one size fits all’ strategy. This causes excessive use of broad-spectrum antibiotics and limited resources. The IntelliSep test is an investigational, in vitro host response test that quantifies the state of immune activation by measuring the biophysical properties of leukocytes. The test is performed on EDTA-anticoagulated blood and results in under 10 minutes. The result, the Intellisep Index (ISI), ranges 0.1-10.0, stratified into three interpretation bands of increasing disease severity risk: Green, Yellow, and Red.

This study assesses the Intellisep Index (ISI) as a potential aid for risk stratification and resource allocation for the treatment of patients presenting to the emergency department with possible sepsis.

**Methods:**

Adult patients presenting to the ED with signs or suspicion of infection were prospectively enrolled at multiple US sites (Apr. – Sept. 2019). Patients were followed by retrospective chart review for outcome information and Sepsis-3 adjudication. Treating clinicians were blinded to ISI results.

**Results:**

290 study patients (sepsis prevalence 17.5%) were stratified by the ISI as 160 (55%) Green, 70 (24%) Yellow, and 60 (21%) Red. The diagnostic odds ratio for sepsis was 20.5 (8.2 - 36.0, 95% CI) between the Red and Green Bands. Subjects in the Green Band had significantly lower mortality (p < .01) and severity of illness, as measured by APACHE II (p < .01), SOFA (p < .001), ICU admission (p < .001) and hospital length of stay among survivors (p < .001). However, 16 (10%) subjects in the Green Band received antipseudomonal and / or anti-MRSA antibiotics in the ED; only two of these subjects were septic (both survived without needing ICU care with 0 and 6-day hospital stays). Conversely, 12 septic subjects in the Red Band did not receive broad-spectrum anti-MRSA antibiotics in the ED, with an average hospital length of stay of 8 days and one fatality.

**Conclusion:**

The ISI, a rapid, quantitative measure of immune activation, may offer ED clinicians an aid for rapid risk stratification of patients presenting with suspicion of infection and guide appropriate treatment.

**Disclosures:**

**Roya Sheybani, PhD**, Cytovale: Employee|Cytovale: Stocks/Bonds **Henry T. Tse, PhD**, Cytovale: Stocks/Bonds **Ajay M. Shah, PhD**, Cytovale Inc: Board Member|Cytovale Inc: Ownership Interest|Cytovale Inc: Stocks/Bonds.

